# Heart Involvement in Multisystem Inflammatory Syndrome, Associated With COVID-19 in Children: The Retrospective Multicenter Cohort Data

**DOI:** 10.3389/fped.2022.829420

**Published:** 2022-03-02

**Authors:** Mikhail M. Kostik, Liudmila V. Bregel, Ilia S. Avrusin, Olesya S. Efremova, Konstantin E. Belozerov, Elena A. Dondurei, Tatiana L. Kornishina, Eugenia A. Isupova, Natalia N. Abramova, Eugeniy Yu Felker, Vera V. Masalova, Andrey V. Santimov, Yuri A. Kozlov, Alexander O. Barakin, Ludmila S. Snegireva, Julia Konstantinova, Alla A. Vilnits, Maria K. Bekhtereva, Vera M. Argunova, Alla E. Matyunova, Polina A. Sleptsova, Tatyana E. Burtseva, Vladimir V. Shprakh, Tatyana V. Boyko, Olga V. Kalashnikova, Vyacheslav G. Chasnyk

**Affiliations:** ^1^Hospital Pediatry Department, Saint-Petersburg State Pediatric Medical University, Saint Petersburg, Russia; ^2^Irkutsk State Medical Academy of Postgraduate Education, Branch of Russian Medical Academy of Continuous Professional Education, Irkutsk, Russia; ^3^Irkutsk Regional Children's Hospital, Irkutsk, Russia; ^4^Scientific Research Institute of Influenza n.a. A.A. Smorodintsev, Saint Petersburg, Russia; ^5^Children's City Clinical Hospital # 5 n.a. N.F. Filatov, Saint Petersburg, Russia; ^6^Pediatric Research and Clinical Center for Infection Diseases, Saint Petersburg, Russia; ^7^Republic Hospital #1–National Center of Medicine, Yakutsk, Russia; ^8^Department of Pediatrics and Pediatric Surgery, North-Eastern Federal University (NEFU), Yakutsk, Russia; ^9^Yakut Research Center of Complex Medical Problems, Yakutsk, Russia

**Keywords:** multisystem inflammatory syndrome, myocarditis, children, hypercytokine syndrome, cytokine storm syndrome, shock, coronary artery lesions, troponin

## Abstract

**Objectives:**

Heart involvement in multisystem inflammatory syndrome associated with COVID-19 in children (MIS-C) is a new challenging problem, requiring fast and reliable diagnostics and appropriate treatment. The aim of this study is to describe heart involvement in patients with MIS-C.

**Study Design:**

In this retrospective, multicenter cohort study, data of 122 patients were included. All patients met WHO and CDC criteria of MIS-C.

**Results:**

Various types of heart involvement in MIS-C patients were observed. Patients with solely coronary artery lesions (CAL, *n* = 10, 8.2%) had typical features of Kawasaki disease: younger age, thrombocytosis and normal ferritin level, without giant CA aneurysms, thrombosis, myocardial infarction, shock, and ICU admission. Patients with solely myocardial involvement (MI, *n* = 30, 24.6%) had an older onset age, elevated ferritin, LDH, the highest D-dimer, H score, and thrombocytopenia level. The following clinical signs were associated with MI: gastrointestinal and central nervous system disorder, sore throat, swelling face, splenomegaly, shock, and treatment in the intensive care unit required. Patients with a combination of CAL and MI (*n* = 10, 8.2%) had symptoms similar to patients with solely MI, except for impressive thrombocytopenia. Shock and ICU admission were found in 34.7% of patients without heart involvement (*n* = 72, 59%). One major criterion [troponin > 32 pg/ml (52 points)] or at least two minor criteria [face swelling (32 points) and D-Dimer > 1,300 ng/ml (29 points)] were associated with MI (>32 points) with a sensitivity of 67.5% and a specificity of 88.9%.

**Conclusion:**

The above-suggested criteria can be added to routine diagnostic procedures to confirm MI in MIS-C patients.

## Introduction

In December 2019, an outbreak of a new coronavirus disease associated with the severe acute respiratory syndrome (SARS-CoV-2) was reported in Wuhan, Hubei Province, China. Since then, the COronaVIrus Disease 2019 (COVID-19) pandemic has rapidly developed into a global health emergency all over the world. According to systematic reviews, cases of COVID-19 in children are observed relatively less frequently (from 4 to 17% of the total number of COVID-19 cases according to various researchers) and generally have a milder course (1–4). However, since April 2020, there has been an increase in the number of children and adolescents with an acute multisystem inflammatory condition that fully or partially meets the criteria for Kawasaki disease (KD), further named a multisystem inflammatory syndrome in children (MIS-C) (5).

These cases were associated with a positive PCR result for SARS-CoV-2, with pronounced gastrointestinal symptoms and progressing toward multiple organ damage, signs of macrophage activation syndrome, heart involvement, and severe shock requiring hospitalization in the intensive care unit (6–9). Most researchers note that MIS-C occurs in older children than KD. It is also often manifested by gastrointestinal symptoms (diarrhea, abdominal pain, and vomiting) and heart damage (myocarditis and pericarditis), often leading to myocardial damage and shock, while these clinical manifestations are less common in KD (10, 11). Many investigators describe signs of cardiac damage, including a significant increase of troponin level, and left ventricular dysfunction in patients with MIS-C (12, 13). According to the study including 286 patients with MIS-C in European countries, heart damage is highly common in these patients, often accompanied by the high level of N-terminal pro-B-type natriuretic peptide, ferritin, D-dimers, and cardiac troponin in addition to elevated CRP and procalcitonin level and worsening of systolic myocardial function in 34% of patients at the time of admission to the hospital, with rapid recovery within a few days of treatment; coronary dilatation was moderate and occurred in 1/5 of the patients (14). The cause of heart damage in MIS-C is not completely clear—the alleged mechanisms may be direct viral damage or an immuno-mediated mechanism or microthrombovasculitis phenomena (15). The data about heart damage in MIS-C are quite limited. So, attempts to determine significant indicators of myocardial injury for possible clarification of its pathogenetic mechanisms are of interest.

The aim of our study was to find clinical and laboratory predictors of myocardial involvement in patients with MIS-C.

## Materials

In this retrospective, multicenter cohort study, data of 122 patients (69 boys and 53 girls) with a median age of 8.9 (5.3; 11.8) years were included. All patients met WHO and CDC criteria of MIS-C (16, 17). We collected the following medical information: epidemiological data showing the presence of COVID-19 disease, method of its identification (PCR throat or nasal swab, IgM, and IgG), family or close contact, time of manifestation after COVID-19 and MIS-C, demographics (age and sex), and clinical features [highest recorded temperature; duration of fever; involvement of gastrointestinal (GI) system, central nervous system (CNS), respiratory system, and cardiovascular system; presence of sore throat; rash; conjunctivitis; red, dry, cracked lips; bright mucosa; cervical lymphadenopathy; distal extremities disorders; peeling of fingers; face swelling; hepatomegaly; splenomegaly; and presence of arthritis]. The following laboratory values were captured: complete blood count, erythrocyte sedimentation rate (ESR), alanine aminotransferase (ALT), aspartate aminotransferase (AST), total protein, albumin, ferritin, lactate dehydrogenase (LDH), C-reactive protein (CRP), triglycerides, creatinine, troponin I, fibrinogen, and D-dimer. Treatment options including transfer to intensive care unit (ICU) were also considered.

Myocardial involvement was detected if there were cardiac arrhythmias, electrocardiography (ECG), and echocardiography (EchoCG) abnormalities, and positive troponin I and/or pro-BNP test. Coronary artery lesions are defined on EchoCG. The laboratory parameters extracted from the medical records were those obtained on the peak of the disease (highest or lowest values), usually between the 7th and 10th days of the MIS-C.

Cytokine storm was diagnosed based on HScore, presence of hemophagocytic lymphohistiocytosis (HLH) according to HLH-2004 criteria, or presence of macrophage activation syndrome, according to 2005 and 2016 Ravelli's criteria (18–21).

### Ethics

Approval of the local ethics committee was not required since we used data from clinical charts. All patients were appropriately anonymized. All patient's representatives and 15 years old and older patients gave consent in their case report form allowing the use of the medical information anonymously.

### Statistics

Sample size was not calculated initially. Statistical analysis was performed with the software STATISTICA, version 10.0 (StatSoft Inc., USA). All continuous variables were checked by the Kolmogorov–Smirnov test, with no normal distribution identified. Continuous variables are presented as median and interquartile ranges (IQRs). Categorical variables are presented as proportions. Missing data were not imputed or included in the analyses. Pearson's χ^2^ test or Fisher's exact test in the expected frequencies <5 was used to compare the categorical variables. A comparison of two and more than two quantitative variables was carried out using the Mann–Whitney test and Kruskal–Wallis ANOVA test, respectively. The ability of each variable to discriminate patients with MI from patients without MI was evaluated with sensitivity and specificity analysis, AUC-ROC (area under the receiver operating characteristic curve) with 95% confidence interval (CI), and calculating odds ratio (OR) for detection of the best cutoffs of continuous variables. The higher values of OR of variables interfere with the better discriminatory ability. We used the “best” threshold for our data's ROC curve analysis because it provides the most appropriate mean between sensitivity and specificity. *p* < 0.05 was considered statistically significant. By univariate analysis, each of the variables of interest was associated with the MI in MIS-C patients, with a *p* < 0.05. They were therefore included in a multivariate logistic model to assess their independent contribution to the outcome. Binary variables included in the model (e.g., face swelling) were coded as present or absent. The threshold value was based on a receiver operating characteristic (ROC) curve analysis, retaining the value at which sensitivity plus specificity was maximized. No interaction terms were included in the model. The pseudo *R*^2^ statistic was used for assessing the goodness of fit of the model. The coefficients resulting from this multiple logistic regression analysis were used to assign score points for the construction of the diagnostic score (Dscore). For each variable that was significantly associated with the outcome in the logistic regression, the rule was to multiply the beta value for each range by 100 and round off to the nearest integer.

## Results

### Epidemiology of COVID-19 in the Patients With MIS-C

COVID-19 was identified by throat/nasal swabs SARS-CoV-2 PCR in 14 (11.4%) at the time of hospitalization due to MIS-C; both IgM and IgG antibodies against SARS-CoV-2 virus were positive in 29 (23.6%), as well as only IgG in 105 (85.4%). Close family contacts were reported in 49 (39.9%). Thirty-one patients (25.2%) had symptomatic COVID-19 infection after family or school contacts, which were confirmed clinically, epidemiologically, and by PCR testing. Patients had mild to moderate fever (*n* = 28, 90.3%), anosmia (*n* = 25, 80.7%), sneezing (*n* = 19, 61.3%), and coughing (*n* = 12, 38.7%). The median time between COVID-19 infection manifestation or close family contact and MIS-C onset was 30.0 (21.0; 40.0) days, and the time between MIS-C onset and hospital admission was 5.0 (3.0; 8.0) days.

### Differences in the Clinical, Laboratory, and Instrumental Presentations in MIS-C With Different Subsets of Patients Based on Heart Involvement

Based on heart involvement, patients were divided into 4 subgroups: (i) patients who had solely coronary artery lesions (CAL, *n* = 10), (ii) patients with solely myocardial involvement (MI, *n* = 30), (iii) patients with a combination of CAL and MI (*n* = 10), and (iv) patients without evident heart involvement (*n* = 72).

Among clinical signs, there were only differences in the frequency of sore throat, peeling of fingers, face swelling, splenomegaly, and shock, as well as the onset age and ICU admissions. In addition, some patients met HLH2004 and MAS2005 Ravelli's criteria. Thus, clinical signs were unreliable as predictors of heart involvement. Among laboratory abnormalities, there were differences in the level of hemoglobin, platelets, ferritin, protein, creatinine, LDH, D-dimer, and Hscore (Table 1).

**Table 1:** Clinical, laboratorial, and instrumental findings in different subsets of the patients with MIS-C according to heart involvement.

**Parameter**	**CAL (*n* = 10)**	**MI (*n* = 30)**	**CAL+MI (*n* = 10)**	**No heart involvement (*n* = 72)**	***p***
**Demographics**					
Age, years	1.9 (1.1; 3.2)	9.5 (4.0; 11.4)	10.2 (9.0; 12.8)	8.9 (5.9; 12.4)	0.0007
Gender, male, *n* (%)	7 (70)	18 (60)	5 (50)	39 (54.2)	0.748
**COVID-19 identification**					
PCR, *n* (%)	1/9 (11.1)	5 (16.7)	2/9 (22.2)	6/69 (8.7)	0.525
IgM, *n* (%)	2/9 (22.2)	8/28 (28.6)	4/9 (44.4)	15/48 (31.3)	0.761
IgG, *n* (%)	9/9 (100)	26/27 (96.3)	9/9 (100)	61/66 (92.4)	0.617
Family contact, *n* (%)	3/8 (37.5)	16/23 (69.6)	5/8 (62.5)	25/43 (58.1)	0.451
COVID-19 clinical signs, *n* (%)	4/6 (66.7)	6/10 (60)	3/4 (75)	18/23 (78.3)	0.737
**Clinical signs**					
GI symptoms, *n* (%)	7 (70)	27 (90)	7 (70)	49/70 (70)	0.187
Neurological symptoms, *n* (%)	4 (40)	16/29 (55.2)	5/9 (55.6)	28/70 (40)	0.489
Sore throat, *n* (%)	5 (50)	21 (70)	10 (100)	40/69 (58)	0.046
Rash, *n* (%)	10 (100)	20 (66.7)	8 (80)	55/68 (80.9)	0.137
Conjunctivitis, *n* (%)	8 (80)	25/28 (89.3)	8 (80)	56/67 (83.6)	0.842
Dry cracked lips, *n* (%)	6 (60)	17/25 (68)	6 (60)	33/68 (48.5)	0.383
Bright mucous, *n* (%)	6 (60)	20/28 (71.4)	8 (80)	34/49 (69.4)	0.804
Respiratory signs, *n* (%)	5 (50)	19 (63.3)	5 (50)	29 (40.9)	0.230
Cervical lymphadenopathy, *n* (%)	6 (60)	23/26 (88.5)	9 (90)	49/69 (71)	0.135
Hands/feet erythema/edema, *n* (%)	7 (70)	22/27 (81.5)	7/8 (87.5)	39/68 (57.4)	0.075
Peeling of fingers, *n* (%)	6 (60)	17/26 (65.4)	3/7 (42.9)	22/66 (33.3)	0.031
Face swelling, %	3 (30)	21/29 (72.4)	6/8 (75)	26/66 (39.4)	0.006
Hepatomegaly, *n* (%)	7 (70)	25 (83.3)	7 (70)	41/66 (62.1)	0.226
Splenomegaly, *n* (%)	2 (20)	21 (70)	4 (40)	17/65 (26.2)	0.0004
Arthritis/arthralgia, *n* (%)	0 (0)	6 (20)	3/9 (33.3)	12/68 (17.7)	0.293
Shock/hypotension, *n* (%)	0 (0)	20 (66.7)	6 (60)	25 (34.7)	0.0005
ICU admission, *n* (%)	0 (0)	25 (83.3)	5 (50)	25 (34.7)	0.000002
Duration of fever, days	12 (7; 17)	11 (8; 15)	9 (9; 13)	10 (7; 13)	0.720
KD criteria fulfillment • Complete, *n* (%) • Incomplete, *n* (%)	6 (60) • 4 (40)	17 (56.7) • 6 (20)	7 (70) • 2 (20)	38 (52.8) • 6 (8.3)	0.026
**Laboratorial**					
Hemoglobin, g/L	98 (88; 103)	98 (86; 113)	113 (109; 123)	107 (97; 114)	0.008
White blood cells, 10^9^/L	16.7 (7.9; 21.1)	16.3 (11.7; 23.0)	16.9 (10.4; 21.7)	15.8 (11.4; 20.0)	0.958
Platelets, 109/L	664 (264; 903)	87 (70; 451)	227 (150; 450)	204 (117; 443)	0.011
ESR, mm/h	57 (40; 67)	46 (36; 52)	43 (22; 53)	44 (30; 55)	0.520
C-reactive protein, mg/dl	8.2 (2.1; 14.5)	14.5 (11.0; 24.2)	18.4 (12.7; 24.2)	13.8 (3.1; 27.5)	0.271
Ferritin, μg/L	101.1 (86.8; 258.4)	417.5 (200.0; 902.2)	348.5 (272.1; 427.5)	180.9 (65.5; 474.2)	0.010
ALT, IU/L	28.2 (16.1; 73.6)	45.8 (25.0; 81.5)	44.5 (21.0; 64.0)	37.5 (23.0; 71.1)	0.750
AST, IU/L	24.5 (21.0; 80.8)	56.0 (39.6; 94.0)	44.6 (31.9; 74.0)	47.0 (30.0; 76.0)	0.217
Serum protein, g/L	61.3 (58.0; 75.3)	50.6 (44.0; 58.0)	61.0 (51.0; 63.9)	58.6 (52.6; 63.7)	0.005
Albumin, g/L	33.1 (28.4; 36.6)	31.0 (25.8; 34.0)	33.4 (24.2; 36.5)	29.5 (26.7; 33.8)	0.663
Triglycerides, mmol/L	1.8 (1.7; 2.5)	2.3 (1.7; 3.6)	2.5 (2.3; 2.7)	2.5 (1.7; 2.9)	0.905
Creatinine, mmol/L	37.6 (36.0; 46.6)	54.0 (41.7; 88.4)	50.0 (49.3; 67.0)	59.7 (46.5; 69.0)	0.020
LDH, IU/L	247.0 (211.0; 274.5)	403.5 (260.0; 597.2)	329.0 (213.0; 382.0)	470.0 (291.0; 663.0)	0.008
Fibrinogen, g/L	4.0 (3.3; 5.4)	3.8 (1.5; 5.8)	5.5 (4.7; 7.7)	4.2 (2.7; 6.2)	0.172
D-dimer, ng/ml	1,335 (583; 2,376.5)	2,420 (1,800; 3,778)	2,471 (1,555; 2,640)	882 (552; 2,000)	0.0008
Troponin, pg/ml	0.0 (0.0; 0.3)	56.0 (3.1; 172.0)	7.9 (5.5; 99.9)	2.5 (1.0; 6.0)	0.011
Hscore	96 (68; 119)	142 (106; 168)	106 (91; 112)	91 (68; 121)	0.0002
**Treatment and outcomes**					
IVIG treatment, *n* (%)	7 (70)	17/28 (60.7)	8 (80)	29/67 (43.3)	0.063
Acetylsalicylic acid, *n* (%)	9/9 (100)	16/29 (55.2)	7 (70)	39/67 (58.2)	0.080
Corticosteroids, *n* (%)	5 (50)	25 (83.3)	10 (100)	53 (79.1)	0.042
Biologics, *n* (%)	1 (10)	3 (10)	0 (0)	0 (0)	0.037
Stay in hospital, days	18 (15; 21)	26 (23; 41)	26 (22; 43)	17 (13; 24)	0.00001

*GI, gastrointestinal; ICU, intensive care unit; ESR, erythrocyte sedimentation rate; ALT, alanine aminotransferase; AST, aspartate aminotransferase; LDH, lactate dehydrogenase; IVIG, intravenous immunoglobulin; CAL, coronary artery lesions; MI, myocardial involvement*.

Subgroup analysis showed that patients with solely CAL had typical features of Kawasaki disease: younger age, thrombocytosis, and normal ferritin level. No giant CA aneurysms, thrombosis, myocardial infarction, shock, and ICU admissions in this group were observed. Furthermore, after implementation of Kawasaki disease criteria, 6 patients (60%) met the criteria of complete Kawasaki disease and 4 (40%) met the criteria of incomplete Kawasaki disease, but all had laboratory (PCR or IgM or IgG) and epidemiological (close family contacts) confirmation of COVID-19 disease.

Patients with solely MI had, on the contrary, distinctive features, like older onset age, thrombocytopenia, elevated ferritin, LDH, the highest level of D-dimer, and Hscore. The following clinical signs were frequently associated with MI: GI and CNS involvement, sore throat, swelling face, splenomegaly, shock, and ICU admission. Seventeen patients (56.7%) with MI met the criteria of complete and six (20%) met the criteria of incomplete KD.

It is notable, that patients with a combination of CAL and MI had symptoms similar to patients with solely MI, with the exception of impressive thrombocytopenia and not so high Hscore. Seven patients (70%) met the criteria of complete and two patients (20%) met the criteria of incomplete Kawasaki disease, and one patient did not meet Kawasaki disease criteria, but with the same confirmation of COVID-19. Patients with MI with and without CAL had differences only in the level of hemoglobin (*p* = 0.008) and Hscore (*p* = 0.035), and frequency of ICU admissions (*p* = 0.035).

Patients without heart involvement had some differences compared to patients with CAL, having more similarity to patients with MI, with the exception of ferritin level and the lowest D-dimer. It is worth mentioning that shock and ICU admission were found in 34.7% of patients without heart involvement. Thirty-eight patients (55.9%) met complete KD criteria and six (8.3%) met incomplete KD criteria. Parameters of MIS-C based on the presence of different types of heart involvement are shown in Table 1.

### Risk Factors of Myocardial Involvement

**Figure 1:**
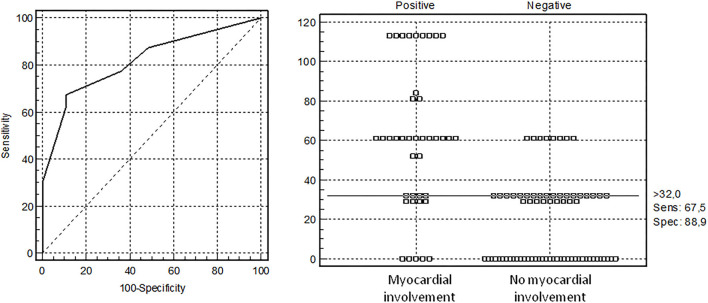
Receiver operating characteristic (ROC) curve analysis for diagnosis of myocardial involvement (MI) in children with multisystem inflammatory syndrome computed with the developmental data set. The optimal cutoff was selected as the threshold giving the highest value for the sum of sensitivity and specificity. Area under the curve (AUC) = 0.818 (0.733–0.884), the threshold for MI >32 points with sensitivity 67.5% and specificity 88.9%.

After exclusion of patients with solely CAL, we calculated risk factors of the MI comparing MI patients with and without CAL (*n* = 40), and patients without heart involvement (*n* = 72). Patients with MI often had sore throat (77.5 vs. 58%, *p* = 0.039), cervical lymphadenopathy (88.9 vs. 71%, *p* = 0.038), hands/feet erythema/edema (82.9 vs. 57.4%, *p* = 0.010), face swelling (73 vs. 39.4%, *p* = 0.001), splenomegaly (62.5 vs. 26.2%, *p* = 0.0002), shock/hypotension (65 vs. 34.7%, *p* = 0.002), and ICU admission (75 vs. 34.7%, *p* = 0.00004). The laboratory assessments in patients with MI showed higher troponin [38.0 (5.0; 172.0) vs. 2.5 (1.0; 6.0) pg/ml, *p* = 0.0007], ferritin [391.0 (261.0; 700.0) vs. 180.9 (65.5; 474.2) μg/L, *p* = 0.003], D-Dimer [2,471 (1,800; 3,577) vs. 882 (552; 2,000) ng/ml, *p* = 0.00006], and Hscore [131 (100; 165) vs. 91 (68; 121), *p* = 0.0001], and lower serum protein [53.0 (44.0; 62.8) vs. 58.6 (52.6; 63.7) g/L, *p* = 0.023]. Factors associated with MI were calculated with AUC-ROC analysis for continuing variables and analysis of sensitivity and specificity (Table 2). Parameters with highest sensitivity, specificity, odds ratio, and clinical meaningful were included in the multivariate analysis. In the multivariate analysis, only 3 variables (troponin > 32 pg/ml, D-Dimer > 1,300 ng/ml, and face swelling) from the initial 15 included in the model remained significantly associated with the probability of being classified as having MI. The optimal cutoff was selected as the threshold giving the highest value for the sum of sensitivity and specificity. The area under the curve (AUC) = 0.818 (0.733–0.884), score for MI > 32 points, allowed to discriminate patients with MI from patients without MI with sensitivity 67.5% and specificity 88.9% (Table 3 and Figure 1). The pseudo *R*^2^ statistic for the model was 0.49 (*p* < 0.00004). The maximum possible scores assigned to each variable were 52 points for troponin > 32 pg/ml, 32 points for face swelling, and 29 points for D-Dimer > 1,300 ng/ml (Table 3). Missing data were scored as 0. According to the analysis, troponin >32 pg/ml was found to be a major criterion, and D-dimer > 1,300 ng/ml and face swelling appeared to be minor criteria. The decision rule is the following: to predict MI in MIS-C, one major criterion or at least two minor criteria are required.

**Table 2:** Factors associated with myocardial involvement in children with MIS-C.

**Parameter**	**Se**	**Sp**	**OR (95% CI)**	***p***
D-dimer >1,300, ng/ml	84.8	63	9.6 (3.1; 29.4)	0.00002
Ferritin >241.0, μg/L	77.1	60.4	5.2 (1.9; 13.7)	0.0007
Hscore >135	47.4	90.3	8.4 (2.9; 24.1)	0.00002
Total protein ≤ 46.1, g/L	35.9	95.9	13.2 (2.8; 62.6)	0.0001
Troponin >31.6, pg/ml	51.9	100.0	-	0.0002
Respiratory signs	59.2	60.0	2.2 (0.98; 4.8)	0.052
Gastrointestinal symptoms	30.0	85.0	2.4 (0.9; 6.7)	0.079
Sore throat	42.0	77.5	2.5 (1.03; 6.0)	0.039
Cervical lymphadenopathy	29.0	88.9	3.3 (1.02; 10.4)	0.038
Hands/feet erythema/edema	42.6	82.9	3.6 (1.3; 9.8)	0.010
Face swelling	60.6	73.0	4.2 (1.7; 10.0)	0.001
Splenomegaly	73.8	62.5	4.7 (2.0; 11.0	0.0002
Shock/hypotension	65.3	65.0	3.5 (1.6; 7.9)	0.002
ICU admission	65.3	75.0	5.7 (2.4; 13.4)	0.00001
MAS 2005	69.4	52.6	2.5 (1.1; 5.8)	0.029

*Se, sensitivity; Sp, specificity; OR, odds ratio; CI, confidence interval; ICU, intensive care unit; MAS, macrophage activation syndrome*.

**Table 3:** Variables included in the development of the diagnostic set and diagnostic score calculation.

	***B***	**SE**	***p***		No. of points (criteria for scoring)^*^
Troponin >32 pg/ml	0.52	0.12	0.0001	Major criterion	0 (<32.0 pg/ml) or 52 (≥32.0 pg/ml)
Face swelling	0.32	0.14	0.024	Minor criteria	0 (no) or 32 (yes)
D-dimer >1,300 ng/ml	0.29	0.13	0.028		0 (≤ 1,300 ng/ml) or 29 (>1,300 ng/ml)

*Diagnostic rule: decision rule is as follows: to predict MI in MIS-C, one major criterion or at least a combination of 2 minor criteria is required.*

* *Score cutoff > 32 points*.

## Discussion

Heart damage in multisystem inflammatory syndrome is one of the dominant manifestations of the disease. In our study, the signs of heart involvement were identified in 50 (41.9%) MIS-C patients with the most prominent myocardial involvement and shock/arterial hypotension. Three criteria were associated with MI (face swelling, troponin > 32 pg/ml, and D-Dimer > 1,300 ng/ml) as well as signs of hemophagocytic lymphohistiocytosis. Since the beginning of the SARS-CoV-2 epidemic, myocardial damage in the acute period of this infection has been described in the literature with increasing frequency. Clinical signs of fulminant myocarditis, a decrease in the left ventricular (LV) ejection fraction or hypokinesia of its walls, and/or a high level of biomarkers (troponin I/T, creatine kinase-MB) were reported (22–24).

According to Babapoor-Farrokhran et al. in 5 out of 68 (7.3%) patients who died from COVID-19, the cause of death was myocardial damage and circulatory failure (25). Some authors reported that heart involvement in MIS-C occurs with a frequency of 33 to 67% (26, 27).

The main cardiovascular clinical manifestations in MIS-C are shock, arrhythmias, pericardial effusion, and dilation of the coronary arteries (14). An increase in the level of pro-BNP, D-dimer, and troponin in combination with elevated CRP and procalcitonin is also very common (14).

Despite the similarities of this syndrome with Kawasaki disease, the nature of heart damage in these two conditions has many differences. Unlike Kawasaki disease, in MIS-C, coronary artery lesions are identified less frequently than myocardial damage (increased troponin blood levels and/or LV dysfunction). The prevalence of myocardial injury in MIS-C over the frequency of coronary dilation is noted by many authors (9, 10, 28–30).

In spite of the severe condition of patients with MIS-C due to multi-organ failure and a significant increase in inflammatory biomarker level, deaths are quite rare (7, 31–34), and the resolution of symptoms occurs in a short time (32, 35). In 20 children hospitalized in critical condition with MIS-C and acute myocarditis (mean LV ejection fracture = 35%, average troponin level was 269 ng/ml), normalization of myocardial contractility and troponin level occurred during treatment within 2 days on average (36). The rapid resolution of systolic dysfunction with a simultaneous decrease in troponin level indicates the primary role of myocardial “stunning” in the development of myocardial dysfunction with the background of cytokine storm in MIS-C (28).

Thus, heart damage in MIS-C, in comparison to KD, has a similar spectrum of clinical and pathomorphological changes. The difference is primarily a milder course of coronaritis, but a much more severe course of immuno-mediated vasculitis and frequent development of shock.

Taking the presence of coronary dilatation as a starting point for dividing patients into 4 groups, and conducting a multivariate statistical analysis, we found out several patterns.

In group 1 with isolated coronary dilation, there were younger children (average age of 2 years), predominantly boys. Each of the diagnostic symptoms of KD occurred in more than half of the patients, and leukocytosis and thrombocytosis characteristics of KD were noted. There were no cases of shock and admission to the intensive care unit in this group. In fact, this group is indistinguishable from the classic Kawasaki disease, although provoked by the new coronavirus SARS-CoV-2. Among children with multisystem inflammatory syndrome associated with COVID-19, some patients with coronary dilation are indistinguishable from the ones with “classic” Kawasaki disease by age, clinical, and laboratory parameters, except for data on the connection with COVID-19 (PCR/antibodies/close contact). Therefore, we can assume that this is an underdiagnosed group of patients with “classic” KD who had clinical and/or laboratory data associated with SARS-CoV-2.

Group 2, with isolated myocardial injury, on the contrary, included school-age children who frequently had diagnostic symptoms of KD (each symptom in ≥60%). The main differences from group 1 were thrombocytopenia, hypoalbuminemia, and hypoproteinemia, indicating severe hepatic dysfunction, and the highest level of troponin, ferritin, ALT/AST, LDH, and D-dimer. In this group, the level of troponin was the highest, indicating the maximum degree of myocardial damage compared to the other groups. At the same time, in group 2, there was the highest proportion of children with shock/hypotension, requiring treatment in the intensive care unit. It is also notable that the level of LDH and D-dimer, the frequency of splenomegaly, and the Hscore elevation in group 2 were significantly higher than inpatients of the other groups. Thus, in group 2, the severity of the myocardial injury was most serious (in terms of shock frequency and troponin increase), accompanied by the highest indicators of the hemophagocytic syndrome (coagulopathy, hepatic injury, Hscore, and splenomegaly) in comparison with all other groups. High frequency of shock and a high level of troponin can directly and inversely depend on each other, although cardiogenic shock usually shows an increase in troponin, unlike shock of a different etiology. A frequently observed combination of serious myocardial injury with the hemophagocytic syndrome in this group is very important for understanding the pathogenesis of MIS-C. This observation indicates the leading role of proinflammatory cytokines in the genesis of MI in MIS-C, predominantly due to the unique properties of SARS-CoV-2, since this virus directly damages immunocompetent cells (37, 38) provoking strike production of pro-inflammatory cytokines. At the same time, it explains the rapid reverse dynamics of the symptoms of myocardial injury and shock in MIS-C patients. The severity of abnormalities in the main biochemical parameters of homeostasis (increased ALT/AST, low albumin and protein levels, excess of normal creatinine values in individual patients) was maximal in group 2 in comparison with group 1 and indicated multiorgan failure.

The symptoms in patients of this group confirm the conclusion of Valverde et al. that there is a statistically significant correlation between the degree of cardiac and biochemical marker abnormality and the need for intensive care support (14).

Group 3—with MI and CA lesions—combined signs of groups 1 and 2. In this group, as in groups 1 and 2, diagnostic signs of Kawasaki disease were noted with high frequency-−70%, accompanied in some patients with hypertriglyceridemia, coagulopathy, and moderate hyperferritinemia. However, the serum ferritin level in group 3 reached 684 ng/ml in one patient (the cutoff point for matching the hemophagocytic syndrome according to Ravelli criteria) (21). Half of the patients with shock were hospitalized in the ICU, but creatinine and albumin/protein serum levels were within normal ranges or increased in patients in the worst condition. Similar data were recently published, showing a negative correlation between albumin level and treatment in ICU (39). Interestingly, in this group, the average platelet count was normal, and none of the patients had thrombocytopenia below 150 × 10^9^/L, although some had mild thrombocytosis (Table 1).

Facial edema, more common in groups 2 and 3 (groups with maximum signs of MI and Hscore), is most likely caused by severe vasculitis (capillary leak) in combination with hypoalbuminemia [29.0 (25.0; 33.0) vs. 31.0 (28.9; 36.0) g/L (*p* = 0.014) in patients with and without facial edema] and/or acute heart failure (HF) phenomena.

Myocardial damage in MIS-C without coronary dilation is often accompanied by macrophage activation syndrome, and myocardial damage along with coronary artery dilation is usually found in combination with signs suggestive of hemophagocytic syndrome. In children with MIS-C, troponin and D-dimer elevation probably demonstrate the role of microvasculitis in the development of myocardial dysfunction.

Finally, group 4, the biggest and of particular interest, had no documented MI/coronary dilation. Patients of this group, as well as in groups 1–3, demonstrated a quite high frequency of the main diagnostic Kawasaki disease criteria, and one-third of them were treated in the ICU. The children of this group had a high fever and typical MIS-C signs of hyperinflammation according to laboratory data. However, serum ferritin and CRP increased moderately, with a slight D-dimer increase and incompliance with hemophagocytic syndrome diagnostic criteria. Such a complex of clinical and laboratory manifestations of MIS-C was found in most of the patients we examined.

Data of patients with isolated myocardial injury and a combination of MI with dilation of the coronary arteries suggest that the mechanism of myocardial “stunning” against the background of cytokine storm manifestations leads to the pathogenesis of MI in MIS-C, which corresponds to the results of the study by Belhadjer et al. In patients with left ventricular dysfunction/cardiogenic shock and signs of hyperinflammation associated with COVID-19, troponin levels were increased in 100%, LV wall hypokinesis was noted in 88.6% during echocardiography, although LV diameter was normal in 82.9%, and coronary dilation was moderate and detected only in 17%, which makes it possible to consider LV wall edema as the main cause of myocardial dysfunction (28).

At the same time, it is obvious that direct myocardial damage in MIS-C also occurs, although there is almost no pathomorphological evidence of heart damage in MIS-C described in the literature, with the exception of two publications. Dholnikoff et al. reported the death of an 11-year-old previously healthy girl from the family of African immigrants. She died a day after hospitalization with signs of MIS-C, respiratory distress syndrome, and cardiogenic shock (40). Pathomorphologically, acute edema, thickening of the endocardium and myocardium, histological signs of myocarditis, endocarditis, and pericarditis with interstitial and perivascular foci of inflammation and necrosis of cardiomyocytes were detected, and SARS-CoV-2 RNA was extracted from the heart tissues. Inflammatory infiltrates consisted mainly of CD68 macrophages and a small number of CD45+ lymphocytes, neutrophils, and eosinophils. In addition, signs of hemophagocytosis were found in the lymph nodes and spleen, which indicated the severity of cytokine storm. Another publication describes pathomorphological signs of myocarditis (with cellular infiltration and necrosis of cardiomyocytes) and coronaritis in the 2-year-old child with IgM and IgG antibodies to SARS-CoV-2 who died of MIS-C 4 weeks after family contact. In this case, the autopsy revealed histopathological signs of the hemophagocytic syndrome (41).

Our observations, along with the above shown pathomorphological comparisons, indicate that the combination of an infectious process in the myocardium caused by SARS-CoV-2 and the proinflammatory cytokine effect in the development of hemophagocytic syndrome in course of MIS-C is a documented cause of rare fatal outcome in this condition.

### Study Limitations

The main study limitations are the retrospective character of the study with the possibility of bias in the selection of this population, partially missing data, impossibility to have the laboratory data from the same time point, and absence of validated criteria of MIS-C.

## Conclusion

Our study helped to find simple clinical and laboratory signs of myocardial involvement in MIS-C patients, patients hospitalized in infectious or emergency departments with suspicion of MIS-C or exanthema infection or fever of unknown origin. The presence of facial edema is a serious clinical hallmark of myocardial involvement in MIS-C, requiring assessment of D-dimer and troponin, in addition to routine laboratory examination. Patients suspicious of myocardial involvement required cardiologic examination and thorough monitoring of heart damage. Monitoring of patients with a high risk of myocardial involvement may influence the treatment options and outcomes.

## Data Availability Statement

The raw data supporting the conclusions of this article will be made available by the authors, without undue reservation.

## Ethics Statement

Ethical review and approval was not required for the study on human participants in accordance with the local legislation and institutional requirements. Written informed consent to participate in this study was provided by the participants' legal guardian/next of kin.

## Author Contributions

MK, LB, VC, VS, and YK contributed to the conception and design of the study. IA, ED, AM, EI, TK, OE, VM, LS, OK, KB, and AS organized the database. MK and IA performed the statistical analysis. MK, LB, and IA wrote the first draft of the manuscript and had full access to all of the data in the study and takes responsibility for the integrity of the data and the accuracy of the data analysis. IA, MK, LB, and KB wrote sections of the manuscript. All authors contributed to manuscript revision, read, approved the submitted version, have contributed equally to all of the following aspects of the manuscript, conception, acquisition of data, drafting, revising the article, involved in drafting the article or revising it critically for important intellectual content, and approved the final version to be published.

## Funding

The work was supported by Russian Science Foundation grant 20-45-01005.

## Conflict of Interest

The authors declare that the research was conducted in the absence of any commercial or financial relationships that could be construed as a potential conflict of interest.

## Publisher's Note

All claims expressed in this article are solely those of the authors and do not necessarily represent those of their affiliated organizations, or those of the publisher, the editors and the reviewers. Any product that may be evaluated in this article, or claim that may be made by its manufacturer, is not guaranteed or endorsed by the publisher.
